# The pleiotropic effects of erythropoietin in infection and inflammation

**DOI:** 10.1016/j.micinf.2011.10.005

**Published:** 2012-03

**Authors:** Manfred Nairz, Thomas Sonnweber, Andrea Schroll, Igor Theurl, Günter Weiss

**Affiliations:** Department of Internal Medicine I, Clinical Immunology and Infectious Diseases, Innsbruck Medical University, Anichstr. 35, A-6020 Innsbruck, Austria

**Keywords:** Erythropoietin, Anemia, Innate immunity, Infection, *Salmonella*, Iron

## Abstract

Erythropoietin (EPO) is a multi-functional cytokine, which exerts erythropoietic effects but also carries anti-apoptotic and immune-modulatory activities upon binding to two distinct receptors which are expressed on erythroid, parenchymal and immune cells, respectively. Whereas EPO ameliorates hemolytic anemia in malaria or trypanosomiasis and improves the course of autoimmune diseases such as inflammatory bowel disease or autoimmune encephalomyelitis, it deleteriously inhibits macrophage functions in *Salmonella* infection in animal models. Thus, the specific modulation of extra-erythropoietic EPO activity forms an attractive therapeutic target in infection and inflammation.

## 1 EPO as central erythropoietic regulator

**Fig. 1:**
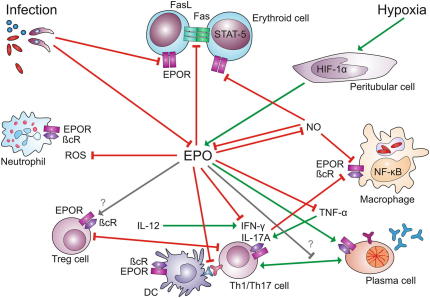
Immune-modulatory effects of EPO: Erythropoietin (EPO) is secreted by peritubular fibroblasts in response to activation of hypoxia inducible factor (HIF)-1α, while nitric oxide (NO) and other pro-inflammatory mediators produced in the presence of infectious agents inhibit renal EPO expression. In erythroid cells, EPO inhibits the elimination of erythroid progenitors by FasL-Fas-induced apoptosis following activation of signal transducer and activator of transcription (STAT)-5. In immune cells, a heterodimeric receptor consisting of an EPO receptor subunit (EPOR) and a β common receptor (βcR) subunit is activated upon binding of EPO. As a consequence, EPO impairs the generation of reactive oxygen species (ROS) by neutrophils and of nuclear factor (NF)-κB-inducible macrophage effectors such as tumor necrosis factor (TNF)-α and NO. Apparently, EPO also converts the functionality of T helper (Th)-1 or Th-17 cells, respectively, to an immune-tolerant phenotype. Whereas EPO stimulates antibody production by plasma cells, its putative effects on other types of immune cells remain elusive. Green arrows indicate stimulatory pathways, red arrows indicate inhibitory pathways, gray arrows indicate putative interactions. (For interpretation of the references to colour in this figure legend, the reader is referred to the web version of this article.)

In its best characterized role, the renal type I cytokine erythropoietin (EPO) acts as key regulator of erythropoiesis. Thereby, EPO inhibits the apoptosis of erythroid progenitor cells by interfering with Fas Ligand (FasL)-Fas signaling, thus enabling terminal erythroid differentiation; mature erythroid cells up-regulate apoptosis-inducing ligands such as FasL and tumor necrosis factor (TNF) related apoptosis-inducing ligand (TRAIL) to suppress the production of immature progenitors by contact-dependent negative feedback mechanisms. These culminate in the cleavage of GATA1, the essential transcription factor in erythroid development, by caspases, resulting in regulated apoptotic elimination of immature erythroid precursors [1,2]. By down-modulating both, FasL and Fas expression (Fig. 1), EPO increases the production of red blood cells (RBCs) and oxygen transport capacity in the circulation [3].

The formation of EPO is induced when peritubular fibroblasts in the kidney sense reduced oxygen tension via oxygen sensitive prolyl hydroxylases (PHDs), which in turn *trans*-activate the key transcription protein hypoxia inducible factor (HIF) [4]. A reduction of hemoglobin (Hb) oxygenation or levels, an increased oxygen consumption in peripheral tissues or a reduced kidney perfusion can induce renal EPO production [5].

However, the kidney is not the only organ which is able to adapt EPO production in response to low oxygen tension. Apparently, chronic hypoxia is also sensed in the epidermis by a HIF-dependent mechanism; nitric oxide (NO)-mediated redistribution of blood flow from inner organs to the skin results in a secondary increase in renal EPO production [6]. Although the kidney is the pivotal production site for EPO, extra-renal EPO formation, for instance in the liver, may also insignificantly contribute to circulating EPO concentrations in adults.

HIF is a heterodimeric transcription factor essential for the control of EPO expression. Whereas the amount of HIF-1α is regulated post-translationally by three PHDs, HIF-1β is constitutively expressed and assumed not to be subject to regulations [7]. Under normoxic conditions, oxygen levels are sufficient for PHDs to continuously hydroxylate HIF-1α at two specific proline residues. Proline hydroxylation, which is modified by concentrations of ferrous iron and 2-oxoglutarate, tags HIF-1α for proteosomal degradation following binding by the von Hippel Lindau (vHL) E3 ubiquitin ligase complex. During cellular hypoxia, in contrast, HIF-1α is stabilized enabling transcription of HIF target genes such as *EPO* and *vascular endothelial growth factor* (*VEGF*). Apart from HIF-1α, two additional isoforms exist. HIF-2α is expressed in renal fibroblasts in response to hypoxia and can dimerise with HIF-1β to drive the expression of target genes including *EPOR* [4,5]. In contrast, due to the lack of a transcriptional activation domain in HIF-3α, this isoform suppresses the expression of hypoxia-responsive genes [4,5].

While HIF-1α and HIF-2α promote EPO expression, nuclear factor (NF)-κB inhibits it [8]. As NF-κB is one of the central transcription factors to initiate and perpetuate inflammation, its negative effect on EPO expression in inflammatory conditions may contribute to the pathogenesis of the anemia of chronic disease (ACD), which frequently occurs in patients suffering from inflammatory diseases thereby negatively impacting on organ function and quality of life [9]. Likewise, via stimulation of NF-κB, interleukin (IL)-1 and TNF-α can inhibit EPO expression *in vitro* [10]. Accordingly, the injection of lipopolysaccharide (LPS) into mice results in reduced expression of *EPO* mRNA in kidneys and decreased levels of circulating EPO [10]. In contrast, the hepatic formation of EPO is stimulated by IL-6 in an NF-κB- and HIF-1α-dependent manner [11], which provides evidence for cross-regulatory interactions between inflammatory mediators and EPO formation (Fig. 1).

Balancing of HIF-dependent gene transcription is of particular importance in compartments with low oxygen tension such as sites of infection and inflammation [12,13]. Most interestingly, NF-κB directly affects HIF-1α expression under hypoxic conditions and induces the accumulation of HIF-1α in macrophages challenged with bacteria, thus linking HIF-1 expression to innate immune response [14].

Furthermore, pharmacological inhibition of PHDs, which initiates HIF-dependent gene expression, efficiently attenuates ischemia-reperfusion injury (IRI) and experimental colitis in animal models [15,16]. However, whether EPO production responds to pharmacological PHD inhibition or to which extent EPO may contribute to the promising tissue-protective and anti-inflammatory effects of these compounds, remains to be investigated in further detail.

In summary, in adults, HIF-regulated renal EPO production provides the essential endocrine signal for the expansion of bone marrow RBC progenitors to compensate for anemia and hypoxia (Fig. 1).

## 2 EPO-inducible signal transduction

A considerable number of studies have investigated EPO-inducible signaling pathways. These data suggest that erythropoietic and tissue-protective effects of EPO are transduced via two different receptors, which do not display functional redundancy [17]. In RBC precursors, the erythropoietic response to EPO is initiated upon binding of picomolar concentrations of this cytokine to EPOR homodimers. This enables binding of proteins containing SRC homology (SH)-2 domains and subsequent activation of Janus kinase (JAK)-2 and of signal transducer and activator of transcription (STAT)-5 [18]. As a consequence, Hb synthesis and cell cycle progression are promoted while apoptosis of erythroid progenitors is inhibited. In parallel to the STAT-5 pathway, EPOR homodimers activate mitogen-activated protein kinase (MAPK) and NF-κB pathways.

**Fig. 2:**
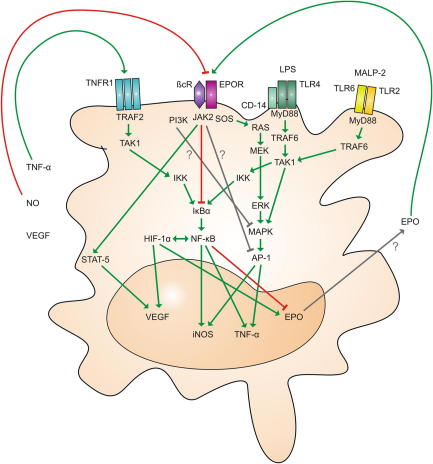
Alternative EPO-induced signaling in immune cells: Macrophage effector functions are regulated by the integration of a broad range of signaling pathways. While toll-like receptors (TLRs) activated via pathogen-derived ligands such as lipopolysaccharide (LPS) and macrophage-activating lipopeptide (MALP)-2 initiate signal transduction via myeloid differentiation primary response gene (MyD)-88 and TNF receptor associated factor (TRAF)-6, binding of (TNF)-α to its receptor (TNFR)-1 activates TRAF-2. Subsequently, transforming growth factor-β-activated kinase (TAK)-1 activates NF-κB via IκB kinase (IKK) and inhibitor of κB (IκB)-α. Binding of EPO to the EPOR-βcR heteroreceptor activates Janus kinase (JAK)-2. JAK-2 is linked to several other pathways including STAT-5 and the RAS-MEK-ERK-MAPK cascade. Phosphoinositide 3-kinase (PI3K) is known to inhibit MAPK, while *EPO* inhibits activator protein (AP)-1 by a yet unidentified mechanism. While NO production is reduced by EPO, NO in turn impairs *EPOR* expression. HIF and NF-κB transcription factors show reciprocal positive interactions, yet have diverse effects on *EPO* transcription since HIF stimulates, while NF-κB inhibits EPO expression. Green arrows indicate stimulatory pathways, red arrows indicate inhibitory pathways, gray arrows indicate putative interactions. Abbreviations: Ras - rat sarcoma; MEK - mitogen-activated protein kinase kinase; MAPK - mitogen-activated protein kinase; ERK - extracellular signal-regulated kinase. (For interpretation of the references to colour in this figure legend, the reader is referred to the web version of this article.)

In cell types other than erythroid progenitors however, EPO utilizes a receptor composed of EPOR and CD131, the beta common receptor (βcR) shared by granulocyte-macrophage colony stimulating factor (GM-CSF), IL-3 and IL-5 (Fig. 2). Although it is still under debate which types of cells express a functional receptor complex [19], the majority of studies suggest that parenchymal, neuronal, immune and neoplastic cells are EPO-responsive [20–22]. Activation of the EPOR-βcR heteroreceptor complex requires high local concentrations of EPO, yet facilitates similar signal transduction events including activation of STAT-5 [23]. However, several other signaling pathways have been implicated in the extra-erythropoietic effects of EPO. These include c-Jun N-terminal kinase (JNK) and phosphatidylinositol 3-kinase (PI3K) activation, respectively, activation of the MAPK pathway and regulation of the binding activity of NF-κB family members (Fig. 2).

While several studies suggest that EPO may stimulate NF-κB binding, we and others have provided evidence that EPO inhibits NF-κB activation; EPO has been reported to sensitize renal carcinoma and myelomonocytic cells to damage by chemotherapeutics due to its ability to interfere with NF-κB activation [24]. Moreover, EPO impairs the formation of pro-inflammatory factors such as TNF-α, IL-6, IL12/IL-23 subunits and NO via inducible NO synthase (iNOS) by macrophages [25]. Mechanistically, theses effects are mediated at the transcriptional level whereby EPO reduces the activation of the NF-κB subunit p65 via the IκB kinase (IKK)-IκB-α pathway, thus lowering the binding activity of NF-κB to its consensus sequences, for instance within the *iNOS* promoter [25]. EPO thus exerts anti-inflammatory effects by inhibiting NF-κB-dependent immune-driven cytokine production which ameliorates the clinical course of experimental colitis while this has been shown to be detrimental in systemic infection with viable *Salmonella* [25]. Interestingly, EPO has been found to activate the NF-κB pathway in the retina causing inhibition of the apoptosis of neuronal cells in response to excytotoxins or IRI [26,27]. Apparently, in different cell types, the spatial organization and temporal sequence of a plethora of mediators produced and pathways involved may dictate which transcription factors are either activated or inactivated by EPO administration in a given signaling context. Relevantly, EPOR homodimers undergo ligand-induced endocytosis and subsequent degradation by a JAK-2-dependent mechanism, which ensures termination of EPO-EPOR signaling thereby regulating circulating EPO levels [28].

A broad range of signaling cascades is initiated after binding of EPO to non-erythropoietic EPOR-βcR heteroreceptors (Fig. 2). Further analyses are required to more precisely work out their regulatory interactions under different physiological and pathologic conditions.

## 3 Extra-erythropoietic functions of EPO

The characterization of rodents carrying genetic defects in the EPO-EPOR system has revealed its essential role for erythropoiesis but also its involvement in other processes in developmental biology such as cardiovascular modeling [4,5]. Deletion of *EPO* or *EPOR* in mice leads to ventricular hypoplasia and angiogenic defects before the onset of embryonic lethality attributable to impaired erythropoiesis [29].

In addition, EPO functions as a tissue-protective and anti-apoptotic cytokine in animal models of IRI, mechanical trauma and organ toxicity at various anatomical locations including the central and peripheral nervous system, retina, myocardium, lung, kidney, pancreas and liver [20,27,30,31].

Apart of its presence on parenchymal cells, the EPOR is also expressed on immune cells. Accordingly, EPO affects immune cell functionality in several *in vitro* and *in vivo* models [32,33].

Specifically, in hemodialysis (HD) patients, EPO has been demonstrated to exert divergent effects on cytokine formation by whole blood cell cultures *in vitro*. While the secretion of IL-2 appears to be increased, TNF-α production is reduced, possibly secondary to increased IL-10 secretion [34].

In experimental autoimmune encephalomyelitis (EAE), a model of multiple sclerosis, EPO inhibits pro-inflammatory responses of antigen-specific T cells and induces immune tolerance [35,36]. By contrast, EPO may promote the clonal expansion and cytokine production by T cells, because the administration of EPO to mice suffering from multiple myeloma stimulates an anti-tumor immune response via CD8^+^ T cells activation [37]. In the same *in vivo* model, EPO-treated myeloma mice show a prolonged survival and a preserved production of polyclonal immunoglobulins by B cells [38]. This EPO-mediated expansion of B cells is associated with an improved antibody response to vaccines [38]. Whether or not treatment with EPO also affects immunoglobulin production in active infectious diseases has not been studied to date [32].

EPO treatment negatively impacts on the production of reactive oxygen species (ROS) in neutrophils (Fig. 1), which may be of relevance for innate immune responses against invading bacteria [39].

Studies with isolated dendritic cells (DCs) have suggested that EPO may have diverse effects on this type of innate immune cells [40]. Specifically, human monocyte-derived DCs express EPOR and treatment with EPO reduces IL-6 but increases IL-12 formation. As increased IL-12 secretion appears to be the dominant feature, DCs show increased effects against myeloma cells after the addition of EPO both *in vitro* and *in vivo* [40].

Similar to DCs, primary macrophages are EPO-responsive. Specifically, murine macrophages express the heterodimeric EPOR and treatment with EPO significantly reduces the formation of NO, TNF-α and cytokines of the IL-6 superfamily [25]. Expression levels of *EPOR* mRNA have not only been investigated in tissue macrophages derived from different organs but also in CD11c^+^ splenic DCs and CD11c^+^, CD4^+^ and CD8^+^ cells isolated from colonic lamina propria of mice suffering from experimental colitis. While splenic and colonic CD11c^+^ DCs also express remarkable amounts of *βcR* and *JAK-2* mRNA, expression of these components of heterodimeric EPOR complexes are relatively low in CD4^+^ and CD8^+^ cells [25]. Thus, EPO may indirectly influence the activity of T cells via alterations in antigen presentation or co-stimulatory signal transduction by antigen presenting cells (APCs).

These anti-inflammatory effects of EPO are in a line with observations in mice showing an ameliorated clinical course of experimental arthritis, colitis and encephalomyelitis following EPO treatment [33,35,36,41]. However, putative protective roles of the intrinsic EPO-EPOR system remain to be characterized. While EPOR expression in the colon is not affected by chemically induced colitis, colonic inflammation results in a significant reduction of local *EPO* mRNA production. It is thus conceivable, that inflammation disrupts an endogenous tissue-protective circuit and that EPO treatment contributes to a restoration of this immune control system [25]. Furthermore, the administration of EPO prolongs the long-term survival of adipose tissue transplants via the combination of pro-angiogenic, anti-apoptotic and anti-inflammatory effects [42]. Therefore, it will be of interest to study whether the extra-erythropoietic actions of EPO may positively affect outcome in solid organ or composite tissue transplantation.

In conclusion, a wide variety of animal studies has delineated the extra-erythropoietic functions of EPO and suggested that it may have therapeutic potential in many conditions including tissue-protection in trauma and ischemia as well as immune-modulation in autoimmunity. However, randomized controlled trials (RCTs) on the use of EPO for purposes other than the treatment of hypo-regenerative anemias remain scarce.

## 4 EPO in infectious diseases

Experimental data on the potential role of the EPO-EPOR system for infectious diseases have emerged recently. EPO has been therapeutically used in infectious diseases to treat pathogen-induced hemolytic anemia. In mice infected with *Plasmodium berghei*, EPO administration does not substantially influence parasitemia, but significantly prolongs the survival. While one might speculate that this is due to amelioration of anemia with EPO treatment, reduced *interferon (IFN)-γ* and *TNF-α* mRNA levels in the brain and diminished neuronal apoptosis imply an anti-inflammatory role of EPO treatment in cerebral malaria [43]. However, it has not been determined which cell types are the mediators of these EPO-mediated tissue-protective effects. Interestingly, cerebral *EPO* mRNA expression is induced during the course of infection, while *EPOR* mRNA declines.

Mice infected with *Plasmodium chabaudi* develop malarial anemia resulting in a compensatory increase of EPO formation in the liver and kidney. However, the amount of EPO produced under these conditions has been considered to be inadequate relative to the degree of anemia. Thus, for correction of malarial anemia, recombinant EPO has been administrated [44]. Upon administration of EPO before the development of anemia, a poor clinical course has been observed. This may be due to EPO-mediated expansion of RBCs thereby promoting pathogen replication whereas EPO may also induce anti-inflammatory effects thereby impairing the immune response towards plasmodia in the circulation. However, when EPO administration is started after parasitemia has plateaued and after the full blown immune response against plasmodia has been initiated, this treatment results in a rapid recovery from malarial anemia and a reduced mortality [44], which may be referred to EPO-mediated inhibition of an overwhelming pro-inflammatory immune response even after pathogen clearance [25].

Contrasting data are available on the role of EPO in mice infected with *Trypanosoma congolense*. While early EPO administration is associated with improved survival due to amelioration of anemia [45], another study has indicated that in three mouse strains infected with the same pathogen, the anemia is insensitive to EPO treatment [46]. However, in acute infection of cattle with *T. congolense*, a compensatory induction of EPO in the kidney and of EPOR in the bone marrow as a feedback response to pathogen-induced anemia has been noted [47].

Data on the role of EPO in bacterial infection are rare. One study has evaluated putative neuro-protective effects of EPO in bacterial meningitis induced in rabbits upon intrathecal application of *Escherichia* (*E.*) *coli* [48]. Although EPO exerts protective effects in non-infectious diseases of the central nervous system [35], the outcome of *E. coli*-meningitis is not influenced by EPO treatment. Whereas analysis has not specifically focused on anti-microbial effectors, it is possible that beneficial cyto-protective effects could be neutralized by the inhibitory effects of EPO towards effector molecules required for efficient elimination of bacteria [48]. Of note, by inhibiting pro-inflammatory immune effector pathways, EPO significantly reduces the survival of mice in a *Salmonella* (*S*.) Typhimurium sepsis model [25]. Accordingly, these effects have not been observed in animals in which the heterodimeric extra-erythropoietic *EPOR* has been knocked out, while the neutralization of endogenous EPO upon application of a specific antibody results in an amelioration of the infection and an improved control of pathogen proliferation [25,49].

The role of EPO administration on the clinical outcome of critically ill patients has been investigated in individuals suffering from systemic infection and receiving appropriate anti-microbial therapy [50,51]. In this setting, EPO administration appears to be safe and is associated with a beneficial clinical course. This corresponds to *in vivo* observations made in rodents in which EPO inhibits immune-driven end organ damage in response to LPS administration, suggesting that the immune-modulatory effects of EPO may be context-dependent [52,53]. However, these data are excellent examples for the pleiotropic effects of EPO in infectious diseases.

In mammals suffering from systemic bacterial infection and not receiving anti-microbial therapy, EPO appears to be detrimental by blocking protective pro-inflammatory immune responses. This can be also of relevance for chronic or latent infections, such as tuberculosis, where weakening of immune control mediated by the TNF-α- and IL-6-family members may promote pathogen growth and disease progression. In contrast, once the pathogens have been killed by antibiotic treatment or after exposure to endotoxin, EPO exerts beneficial effects by down-regulating the overwhelming, sepsis/endotoxin-induced cytokine storm, thereby presumably increasing blood pressure by inhibiting NO formation and limiting radical- and cytokine-mediated tissue damage.

Of interest, the EPO-EPOR system not only controls inflammation but is itself regulated by inflammatory stimuli. For instance, *EPOR* mRNA expression on macrophages is down-regulated by NO, IFN-γ, IL-17A, LPS and *S*. Typhimurium (unpublished observations). The down-regulation of *EPOR* by pro-inflammatory stimuli may result from a feedback mechanism in order to limit the anti-inflammatory effects of EPO under conditions in which these are associated with poor outcome such as in infections. Accordingly, data obtained upon administration of neutralizing EPO-antibodies suggest that physiologically circulating EPO levels adversely affect the protective host defense in *S.* Typhimurium septicemia [25,49].

Moreover, in diabetic chronic kidney disease (CKD) patients endogenous EPO levels are positively correlated with markers of inflammation and are predictive of mortality [54], suggesting that the intrinsic EPO-EPOR system has essential functions in inflammation and infection control *in vivo*.

Whereas *EPO* mRNA is expressed by various types of immune cells, little is known about the formation and secretion of EPO by these cells and how the EPO-EPOR network is affected by pro-inflammatory and anti-inflammatory mediators in inflammation.

Based on the fact that EPO-mediated signals are transduced either via EPOR–EPOR homodimers or through EPOR-βcR heterodimers [17,55], EPO derivatives that selectively initiate either erythropoietic or extra-erythropoietic functions have been developed and studies in inflammatory and infectious diseases have been conducted [56–58]. For example, ARA290 is a novel peptide representing the amino acid sequence of EPO’s helix B, which is thought to selectively interact with the EPOR-βcR heteroreceptor. The effects of ARA290 have also been evaluated in an *in vitro* model of urinary epithelial cell infection where ARA290 co-stimulated the production of the chemokine IL-8 by urinary epithelial cells in response to infection with uro-pathogenic *E. coli* [59].

In a model of polymicrobial peritonitis induced by cecal ligation and puncture, an alternative compound, carbamylated EPO, increases survival which has been traced back to its ability to inhibit TNF-α secretion [60].

## 5 Perspective

The primary physiological function of the EPO-EPOR system is to stimulate bone marrow production of erythroid progenitors in response to renal hypoxemia, thus maintaining an adequate systemic availability of RBCs as oxygen carriers.

Numerous studies have revealed previously less appreciated roles of EPO-EPOR signaling, for instance at sites of tissue damage and inflammation. Administration of EPO exerts disease-modifying effects in animal models of traumatic, ischemic or toxic injury to the nervous, digestive and cardiopulmonary systems. However, the potential therapeutic benefits of EPO therapy in humans are currently outweighed by its primary erythropoietic effects since the expansion of the RBC mass increases the risk of thromboembolic complications and is under certain circumstances associated with an adverse clinical course [9,61]. Thus, EPO derivatives without erythropoietic effects have been developed [56–58].

Recent studies have characterized EPO as a potent anti-inflammatory cytokine in chronic inflammatory disorders and infectious diseases [25,30,36,41]. Specifically, EPO down-regulates pro-inflammatory immune effector pathways in response to chemical tissue damage, LPS stimulation and *Salmonella* infection. However, the expression of anti-inflammatory mediators such as transforming growth factor (TGF)-β, IL-10, IL-27, IL-35 and adiponectin are not regulated by EPO treatment at least in the mouse models investigated so far [25], implying that EPO is a direct anti-inflammatory mediator. In addition, EPO may affect immune cell differentiation and proliferation and it will be of importance to determine such immune-regulatory effects in regard to T cell differentiation or macrophage polarization (M1 versus M2 phenotype) under inflammatory conditions and in cancer models *in vivo* [62].

Although EPO is known to influence several signal transduction cascades, the anti-inflammatory action of EPO towards macrophages has been traced back to inhibition of NF-κB p65 subunit activation and subsequent activation of target gene expression [25]. NF-κB/Rel proteins are crucial transcriptional activators of many pro-inflammatory cytokines such as TNF-α, IL-1β, IL-6, IL-12 and IL-23, chemokines such as monocyte chemoattractant protein (Mcp)-1 and macrophage inflammatory protein (Mip)-1α as well as for host response enzymes including iNOS [63]. Conceivably, NF-κB subunits are key components for immune activation in various inflammatory conditions. In *Pseudomonas* (*P.*) *aeruginosa* pneumonia, for instance, the activation of NF-κB protein p65 is essential for adequate host defense. Whereas over-expression of *p65* promotes TNF-α production and bacterial clearance, blockade of classical NF-κB activation by a dominant negative inhibitor of the IκB α subunit results in increased pulmonary *Pseudomonas* loads [64]. Similarly, inhibition of *p65* with antisense oligonucleotides in *Staphylococcus aureus* sepsis, results in reduced elimination of the microbes from organs [65]. Moreover, mice deficient in the *p50* subunit of NF-κB display increased susceptibility to infection with *Streptococcus pneumoniae* and impaired clearance of *Listeria monocytogenes* [66]. Given the essential role of the NF-κB cascade for host defense, it is tempting to speculate that EPO treatment may be of disadvantage in mice or individuals infected with these pathogens, too.

Of interest, reciprocal interconnections exist between HIF-1α and NF-κB pathways, which act up-stream or down-stream of EPO, respectively. IκB kinase (IKK)-β-dependent activation of NF-κB stimulates *HIF-1α* transcription following infection of macrophages with group A *Streptococcus* or *P. aeruginosa*, as a mechanism to stimulate the production of HIF-induced anti-microbial peptides [14]. Furthermore, HIF-1α activates NF-κB in neutrophils and DCs exposed to hypoxia [12,67,68]. A further interconnection of the HIF and NF-κB pathways is based on the fact that the former induces while the latter represses EPO expression. Interestingly, both HIF and NF-κB activities are modulated by intracellular iron availability [69]. It is currently unknown however, whether the interactions of HIF-1α and NF-κB pathways also affect the EPO-EPOR system in infectious diseases.

As EPO inhibits NF-κB p65 activation, it may have the capacity to interfere with the autocrine and paracrine stimulation of cytokine production by macrophages and other immune cells within inflamed microenvironments, which may potentiate its immune-suppressive effects (Fig. 2). Of relevance, disease-modifying drugs for inflammatory diseases such as glucocorticoids, sulfasalazine and leflunomide act, at least in part, via inhibition of NF-κB functions. Consequently, the addition of EPO or EPO-like peptides acting only on the heterodimeric receptor to such medications may help to accomplish dose reductions, thus limiting toxic side effects associated with current therapeutic regimens. Anti-cytokine strategies, such as application of anti-TNF-α antibodies, have been proven to be effective in autoimmune disorders, such as rheumatoid arthritis or inflammatory bowel disease. As EPO suppresses the production of NO, TNF-α, IL-6 and IL-17 family members via its action on mature immune cells such as macrophages and antigen-stimulated T cells, it may interfere with such pro-inflammatory networks by reducing local and circulating levels of disease-associated cytokines.

The interconnections between EPO activity, iron homeostasis and immune-modulation warrant further notification. In erythroid cells, EPO has been shown to affect iron homeostasis due to its impact on transferrin receptor (TfR)-1 expression [70]. Iron homeostasis is also a key determinant of macrophage functions, on the one hand via promoting ROS formation, while on the other hand iron loading of macrophage inhibits pro-inflammatory, LPS- and IFN-γ-driven immune effector pathways such as *iNOS, TNF-α* or *major histocompatibility complex* (*MHC*) *class II* expression [71–73]. Therefore, it has been considered that EPO’s immune-debilitating effects on macrophage responses may be a consequence of iron retention in macrophages, which would also increase the proliferation of intramacrophage bacteria such as *Salmonella* which are highly dependent on a sufficient amount of iron [74]. Available *in vitro* data suggest that isolated monocytes and macrophages do not respond to EPO with adaptation of iron handling [25,70]. Nonetheless, the systemic effects of EPO on body iron homeostasis and macrophage iron retention, which occurs under inflammatory conditions leading to the development of ACD need to be further evaluated [9]. This is of interest, because EPO and hypoxia have been demonstrated to reduce the expression of the central regulator of iron homeostasis, hepcidin [75,76]. Moreover, macrophages produce minute amounts of hepcidin to control cellular iron egress in an autocrine fashion [77]. However, it will be of interest to study whether EPO may affect the IL-6-inducible production of hepcidin in these cells. Nonetheless, in human subjects treated with EPO, effects on hepcidin formation and body iron homeostasis have been reported [78]. Hepcidin affects macrophage iron homeostasis by blocking cellular iron release leading to retention of the metal within these cells. Of note, hepcidin affects immune responses either secondary based on the negative regulatory effect or iron on IFN-γ-inducible macrophage effector functions [71–73,79], but also via a not yet characterized pathway which leads to inhibition of pro-inflammatory cytokine formation by hepcidin in mice [80,81]. This also translates to a disease-modifying phenotype in a mouse malaria model, which has been traced back to modulation of hepcidin expression [82].

**Fig. 3:**
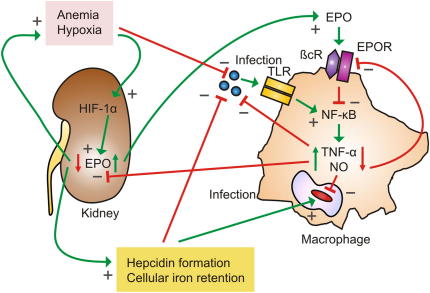
The EPO-immunity regulatory network affects host–pathogen interaction: Microbes induce host response pathways for example via activation of Toll-like receptors (TLRs), thereby leading to stimulation of the NF-κB pathway and subsequent transcription of genes such as *tumor necrosis factor* (*TNF*)-*α* or *inducible nitric oxide* (*NO*) *synthase* (*iNOS*) resulting in formation of the toxic radical NO, needed for effective anti-microbial immune responses. In contrast, after binding to its specific cell surface receptor, EPO inhibits these pro-inflammatory immune effector pathways. Thus, to ensure an effective pro-inflammatory immune response during infection, immune-derived molecules such as TNF-α and NO reduce the anti-inflammatory activity of EPO by down-regulating expression of its cell surface receptor (*EPOR*) and by directly inhibiting EPO formation in the kidney. As a consequence, anemia develops which is due to reduced EPO availability and retention of iron within macrophages mostly based on the action of the acute phase protein hepcidin, whose expression is negatively controlled by EPO. Thus, by these pathways, the availability of oxygen and iron for rapidly growing tissues and/or extracellular microbes is reduced which may be part of the innate immune response, although the access of intracellular pathogens to iron may be contrastingly affected. Oppositely, anemia and hypoxia stimulate EPO formation via activation of hypoxia inducible factor (HIF) in the kidney but maybe also in other cells including macrophages. EPO down-regulates the pro-inflammatory immune response, thereby also reducing the anti-proliferative effects of this cytokine on erythroid progenitor cells. Furthermore, EPO inhibits hepcidin formation, thus enabling mobilization of iron from macrophage stores for erythropoietic use. These events lead to down-scaling of inflammation and correction of anemia after the infection has been cleared by anti-microbial effector pathways. Green arrows indicate stimulatory pathways, red arrows indicate inhibitory pathways. (For interpretation of the references to colour in this figure legend, the reader is referred to the web version of this article.)

In summary, reciprocal regulations of the EPO-EPOR system and inflammation exist (Fig. 3). On the one hand, application of EPO inhibits pro-inflammatory host responses towards certain infectious pathogens, on the other hand, under inflammatory conditions, the EPO-EPOR system is negatively controlled at affected sites. TNBS-colitis causes a substantial decrease in colonic *EPO* mRNA levels whereas splenic *EPOR* expression is significantly down-regulated in response to systemic *Salmonella* infection [25]. In addition, LPS administration reduces EPO formation in the kidney [10]. Therefore, activated immune cells may down-regulate *EPOR* expression in the attempt to evade the immuno-suppressive effects of EPO, presumably as a prerequisite for pathogen control. There is compelling evidence for a deleterious effect of EPO on the course of infections with intracellular pathogens in mice. Whether or not EPO affects immune response pathways and the course of disease in infections with viruses, extracellular bacteria or fungi is currently largely unknown. The influence of EPO on systemic iron homeostasis and hepcidin expression is of major interest in infection, because the availability of iron for pathogens is a hallmark deciding on the fate of many infections [74]. In addition, putative effects of EPO on the functional activity of other immune cells including specific Th subsets, NK and NKT await specific investigations.
